# The day-of-invitation effect on participation in web-based studies

**DOI:** 10.3758/s13428-021-01716-0

**Published:** 2021-10-28

**Authors:** Hans-Georg Wolff, Anja S. Göritz

**Affiliations:** 1 Organizational and Economic Psychology, University of Cologne, Cologne, Germany; 2 Occupational & Consumer Psychology, University of Freiburg, Freiburg im Breisgau, Germany

**Keywords:** Web-based research, Day of the week, Invitation, Response, Retention

## Abstract

Several methods have been proposed to promote participation in web-based research. Here, we examine a technique that is available at no cost: Inviting respondents per e-mail on a particular day of the week. We base our reasoning on such a day-of-invitation effect upon theories on variations in mood and work performance over the week. We conducted five experiments with large and heterogeneous samples to find out whether such effects apply for response rate (i.e., visiting the first page of a study) and retention rate (i.e., completing the study) in web-based studies. We found evidence of a small but significant day-of-invitation effect. Response rate is high at the beginning of the workweek and falls to a low on Friday. Exploratory analyses showed that this decline is higher for employed (vs. nonemployed) persons. Effects on retention rate appear to follow a less straightforward pattern. We discuss possible mechanisms that might account for the day-of-invitation effect and recommend inviting participants on Monday or Tuesday.

**Supplementary Information:**

The online version contains supplementary material available at 10.3758/s13428-021-01716-0.

The WWW has become a widely used survey mode. For example, the market share of web surveys in Germany is 40%, higher than the share of any other mode (ADM, 2019). The bulk of online studies are conducted in online panels (Callegaro et al., 2014) because relying on pre-recruited respondent saves time, money, and methodological challenges compared to recruiting people afresh (Göritz, 2014b).

In view of declining response rates (cf. Lindgren et al., 2020), promoting data quantity in web-based research is important. Hitherto, researchers have tried to influence individuals' likelihood of taking part in a web-based study by, for example, offering them incentives for participation (Göritz & Wolff, 2007; Göritz et al., 2008), varying the field period of studies (Göritz & Stieger, 2009), sending reminders (Göritz & Crutzen, 2012), keeping studies short (Göritz, 2014a), or by building trust in the survey sponsor (Fang et al., 2009).

In the work at hand, we examine whether the day of the week on which individuals are invited to take part in a web-based study influences their likelihood to participate in the study. Proverbial expressions such as “Blue Monday” or “Thank God it's Friday” suggest that a person's psychological state depends on the day of the week. Several strands of research have found evidence for weekday effects on mood and emotions (e.g., Wang et al., 2016), work-related factors (e.g., Bryson & Forth, 2007), risk-taking and poll outcomes (e.g., Sanders & Jenkins, 2016), stock returns (e.g., Birru, 2018), or no-shows to medical appointments (Ellis & Jenkins, 2012). In this vein, we suggest that the day of the week people are sent an invitation to take part in a web-based study influences their decision to participate in the study. Although we do not expect large effects of the day of invitation on participation, taking advantage of small but systematic variations in people's willingness to participate in studies, would be a cost-free and low threshold measure to increase data quantity. Along with other papers published in this journal (e.g., Anwyl-Irvine et al., 2021; Correia et al., 2021; Grootswagers, 2020; Söderström et al., 2021), the present study thus adds to our knowledge on advancing the quality of online studies.

Several studies have examined whether the day of the week affects participation in online surveys. In ad hoc studies, there is some limited evidence that participation is higher in the beginning of the week. Zheng (2011) reports that invitations sent via SurveyMonkey on Mondays attain the highest response rates, which decline over the workweek. Lewis and Hess (2017) found higher participation rates in an employee survey when they sent invitations on Tuesday, as opposed to Wednesday or Thursday. Also, Wednesday, along with Monday and Tuesday, was the best day in Faught et al. (2004). Shinn et al. (2007) as well as Sauermann and Roach (2013) did not find significant differences between weekdays, though the latter reported that Wednesdays and Saturdays seemed to yield lower participation rates.

While studies reported in the previous paragraph used ad hoc samples, Lindgren et al. (2020) examined day-of the week effects in an online panel and thus resembles our study more closely. In online panels, participants have already agreed to participate in survey research and may not perceive invitations as unsolicited e-mails or even spam mails. Lindgren et al. found some differences in response rates after 24 h, but there were no significant differences in response rates between days of the week after 6 days (prior to sending a reminder). Differences in participation rates were small (i.e., ± 2% around the mean) with a high on Wednesdays. When asked, participants stated that they preferred receiving invitations on Monday or Sunday. Moreover, Lindgren et al. used panel data to examine whether day-of-the-week effects varied across different target populations. However, next to a lower participation rate of middle-aged persons on Saturday, the authors do not find any significant differences with regard to age and employment status.

These studies mostly relied upon an exploratory approach, which, despite high sample sizes, might suffer from lower power to detect small effects. Moreover, they rarely ground their findings in theoretical bases that might account for these differences. In this research, we use findings from other research on weekly cycles to argue that linear or other patterns may exist. Our approach remains largely exploratory, but employs theorizing to narrow down potential modelling choices. In the following paragraphs, we will first present an overview of differences in people's feelings and behavior over the days of the week. We will then sketch how these factors might influence participation in Web-based studies.

In contrast to daily, monthly, or yearly cycles that roughly follow solar, lunar, or seasonal rhythms, weekly cycles do not follow any geophysical events but are socially construed (Larsen & Kasimatis, 1990). The week strongly influences the temporal organization of our lives and subsequently our behavior and expectations. Specifically, for Western, mostly Christian countries where we conducted this research, people typically carry out many duties such as going to work or school from Monday to Friday, whereas the weekend allows relaxing from these duties. Similarly, many services (e.g., governmental, medical) have office hours during the week only, and on Sundays shops are closed in many countries with a Christian background. Also, the television program on Saturday and Sunday differs from the program on weekdays.

Two streams of research have examined whether this weekly rhythm influences psychological states. First, several studies have taken the proverbial Blue Monday as a starting point to examine differences in mood across the week. When individuals are asked on which day they believe their mood is worst, they usually choose Monday. The happiest weekdays, by contrast, are thought to be Friday and the weekend (Ellis et al., 2015; Farber, 1953; Stone et al., 1985). However, these beliefs may or may not correspond to people's actual affective experiences when they report their mood on a specific day. Studies have consistently found that weekends, indeed, involve higher levels of good mood and lower levels of bad mood (Clark & Watson, 1988; Csikszentmihalyi & Hunter, 2003; Egloff et al., 1995; Helliwell & Wang, 2014; Kennedy-Moore et al., 1992; Larsen & Kasimatis, 1990; McFarlane et al., 1988; Reis et al., 2000; Rossi & Rossi, 1977; Sheldon et al., 1996; Stone et al., 1985; Tsai, 2019). In contrast to the weekend effect, the majority of the studies do not find a Blue Monday effect; one exception is Reis et al. (2000).

With regard to the online realm, an analysis of blogposts by Mihalcea and Liu (2006) shows that content posted during the week is less positive than on weekends, and overall, blogposts are most negative on Wednesday. A Twitter analysis by Wang et al. (2016) found that tweets referring to linguistic categories of stress, negative emotions, and positive emotions followed a u-shaped pattern, with a low on Fridays for stress and negative emotions, and a low on Tuesdays for positive emotions. They also report that, somewhat inconsistently, the maximum in all three categories is at the weekend – possibly due to nonwork hassles. Furthermore, Taylor's (2006) respondents reported lower mental health on Tuesday, Wednesday, and Thursday, and better mental health when asked Friday to Monday. In a smartphone-based experience sampling study, Stieger and Reips (2019) found self-reported well-being to be low on Sunday evening through Wednesday, but high on Thursday to Saturday. In sum, with slight variations, these studies show that effect tends to be more positive on the weekend and less positive mid-week (i.e., Tuesday to Thursday). There is little evidence of the proverbial Blue Monday.

Second, further evidence for day-of-the-week effects comes from scholarship in the work domain. For example, Bogiss (2000) reports higher absence rates on Mondays and Fridays. Bryson and Forth (2007) examined data from the UK time use survey and found that working time is highest during midweek (i.e., Tuesday to Thursday). Furthermore, Taylor (2006) reports that job satisfaction is lowest on Tuesday, Wednesday, and Thursday and highest on Sunday and Monday. Thus, most empirical results from the work domain follow a curvilinear pattern with a deflection at midweek.

In summarizing existing research, Bryson and Forth (2007) identified three processes that scholars draw upon to explain the curvilinear patterns over the week. First, in line with the effort-recovery model (Meijman & Mulder, 1998), the authors propose that fatigue increases over the week, being lowest on Monday and highest on Friday, because people expend effort at work and have little time to recover at night. In addition, fatigue should decrease on weekends due to the chance to relax and recover from work. Note, however, that empirical evidence is less clear, as studies do find that fatigue is lower during the weekend (e.g., Binnewies et al., 2010; Rook & Zijlstra, 2006), but fatigue appears to increase on Monday and seems to remain constant across the workweek (Rook & Zijlstra, 2006; Weigelt et al., 2021), possibly indicating that further processes may be involved, for example, anticipation, (Weigelt et al., 2021), or re-habituation processes. Second, re-habituation processes entail that individuals’ performance may be lower on Mondays after a weekend off-work as they are out of practice and need to re-habituate to their work patterns. For example, re-habituation has been held responsible for heightened injury rates on Mondays (Brogmus, 2007). Finally, the uplift of individuals' mood towards the end of the week may result in a "final spurt" in work performance on Fridays. Taken together, these processes, among others, might account for the curvilinear relationship between day of the week and various variables.

In the present work, we examine whether similar effects exist on web study participation. Specifically, we suggest that mood and fatigue influence a potential respondent's decision to participate in a web-based study, assuming that re-habituation effects are unlikely in survey participation. Good mood should be positively related to participation, because positive mood promotes helping behavior (Carlson et al., 1988). Therefore, response rates should be lower during midweek and higher on other days. With regard to fatigue, we expect that participants will be less likely to participate when fatigue is comparatively high. With fatigue increasing over the week, participants should be increasingly less inclined to muster the effort to complete an online study. Taken together, if mood and fatigue are mechanisms that translate the effect of the day of the week onto participation behavior, participation should be more likely when sending an invitation on weekends and at the beginning of the week (i.e., Saturday to Tuesday) and less likely when sending an invitation midweek (i.e., Wednesday to Friday).

This being said, it is unlikely that psychological variables alone such as fatigue and mood determine response as a function of the day of invitation. Over the course of the week, the kind and amount of concurrent activities and availabilities fluctuate as well. For example, in theory, Saturday and Sunday might be the best days to send out a study invitation as on the weekend mood is at a high and fatigue at a low. However, a share of invitees might not have Internet access or time to take part in studies while indulging in leisure activities. In consequence, sending a study invitation on the weekend might not achieve the highest response rate after all.

Elucidating the multifactor causality of participation as a function of the day of invitation gets even more complicated because some catching up is possible. To stick with the example, people who do not take part in a study they were invited to on the weekend can nevertheless participate as long as the study is open. The extent of this catching up, however, is limited for two reasons. First, an invitation is likely to achieve the highest impact when it freshly hits the inbox. When inviting pre-recruited people to take part in a web-based study, 70–90% of expectable responses usually occur within 3 days (Batinic & Bošnjak, 2000; Göritz, 2007; Gräf, 2001). Second, letting a few days of field time elapse without taking part in a study reduces the remaining chances to participate because less time is left, and the invitation is sinking in the inbox as fresh messages pour in. According to this reasoning, it appears important on which day a researcher sends out invitations. We will examine whether the day of invitation actually matters.

In the following, we present five experiments from an online access panel that examines the effects of the weekday of the invitation on the response rate and on the retention rate. In going with a meta-analysis on web study participation (Göritz, 2006), we define the *response rate* as the number of invitees who visit at least the first page of a study divided by the total number of people invited to a study. The *retention rate* is the number of responders who stay until the end of a study divided by the number of people who visited the first page of a study. To avoid confounds, in each of the five experiments, no matter on which day the invitation to a given study was sent out the field time of the study was constant, and invitations were sent at the same time of day.

Next to the day of the week, we capitalize on two further opportunities to gain further insights into the day of the week effect. First, in one sample, we chose to invite participants in a week that included a bank holiday in some federal states of Germany but not in others. If the pattern of response as a function of the day of the invitation is different in the resulting natural groups (i.e., with and without a holiday), this would lend support to the notion that there are day-of-invitation effects that are footed in a social construal of time (Larsen & Kasimatis, 1990) rather than in deeply rooted natural rhythmicity. The bank holiday disrupts the socially construed structure of a regular workweek. For example, people may be more likely to go on vacation in a week with a bank holiday.

Second, upon registration online panels usually request some information, such as gender or employment status from participants that is stored in the database. Studies often assess this data and pre-recording reduces the burden of participants and also allows inviting respondents with specific characteristics to studies. As these data are available for non-respondents to subsequent studies in the panel, they may help elucidating reasons for nonresponse. As discussed with regard to the work domain in the previous paragraphs, employment provides a socially construed temporal structure that might affect response rates over the week or weekend. Lindgren et al. (2020) used this strategy when they explored whether age and employment status moderated the relationship between day of invitation and response. While these authors found some effects on response speed (i.e., responding within 24 h), they found few effects after 6 days: Middle-aged persons were less likely to respond on Saturday as compared to Wednesdays. Note, however, that these multiple comparisons may suffer from low power and that only differences within employed and unemployed participants, but not between these groups were examined. With regard to a social construal account, how employment status might affect participation on a given day appears of importance. Upon the suggestion of an anonymous reviewer, we therefore adopt Lindgren et al.’s perspective here, and explore whether panel data collected upon registration moderates the relationship between day of the week and response or retention.

## Materials and methods

### Sample and procedure

We conducted five experiments in a German-speaking opt-in online panel (www.wisopanel.net; see e.g., Göritz et al., 2021). The panel harbors volunteers from all walks of life, who have agreed to participate in web-based studies. Panelists had been recruited into the panel from different sources, for example, via fax, e-mail, flyer, and letter (Göritz, 2004), through links and banners on other websites, word-of-mouth, postings in mailing lists as well as search engines.

In sum, 13,472 participants received an invitation e-mail to participate in at least one of the experiments. Of these, we excluded 596 participants who did not have permanent residence in Germany for a sample size of *N =* 12,876. Note that some participants were repeatedly invited, on average panel members received an invitation to 2.30 studies (*SD* = 0.80, Median = 2). Taking repeated observations into account, we sent 29,592 invitations to the 12,876 panel members. As Table 1 shows, the number of invitations (i.e., observations) ranged from *n*_invited_ = 710 in Study 2 to *n*_invited_ = 11,624 in Study 5. Our sample size for retention is limited to those who responded. With an overall response rate of 30.9% (cf. Table 2), analyses for retention consist of 9135 observations on 5218 individuals.

**Table 1:** Study characteristics

Study	*n*_invited_	Response *n* (%)	Retention *n* (%)	Age M (SD)	Women %	Study duration Median	Reward	Study specifics
1	2752	1522 (55%)	1325 (87%)	34.3 (11.49)	53%	5 min	None	No invitations sent on Friday and Saturday
2	710	592 (83%)	449 (76%)	33.1 (10.50)	51%	9 min	Lottery, 5 prizes 100€ in total	No invitations sent on Tuesday, Wednesday, and Thursday
3	2883	1542 (54%)	1452 (94%)	35.2 (11.45)	53%	5 min	Reward manipulation: Lottery, 3 prizes, 90€ in total for 66% of respondents	No invitations sent on Sunday due to technical problems For *n* = 1532 participants, the week had a bank holiday
4	11,623	3002 (26%)	2963 (99%)	43.0 (13.94)	62%	19 sec	None	--
5	11,624	2477 (21%)	1679 (68%)	43.0 (13.94)	62%	13 min	Per-capita payment	--

**Fig. 1:**
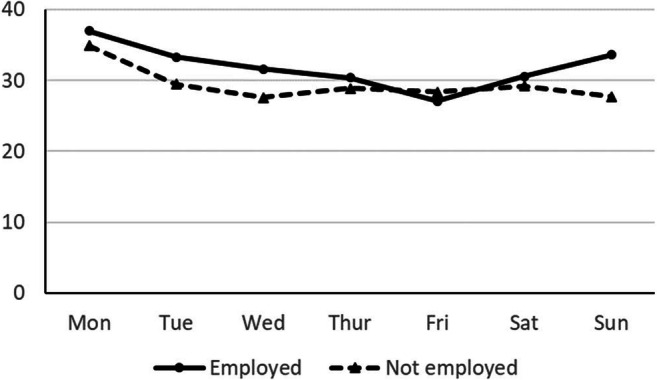
Response rates for employed and nonemployed observations by day of the week

**Table 2:** Response and retention rates in the five studies

Study	Mon	Tue	Wed	Thu	Fri	Sat	Sun	Total
1								
Response	57	61	50	52	--	--	58	55
Retention	87	87	87	87	--	--	87	87
2								
Response	88	--	--	--	83	79	84	83
Retention	76	--	--	--	78	75	75	76
3								
No Holiday week								
Response	56	51	50	51	56	55	--	54
Retention	96	93	92	90	93	92	--	93
Holiday week								
Response	54	57	62	50	51	48	--	54
Retention	93	96	93	98	98	92	--	95
4								
Response	29	24	24	26	23	29	26	26
Retention	99	99	99	98	98	99	99	99
5								
Response	21	23	22	20	21	21	21	21
Retention	68	73	62	71	67	68	67	68
Total^a^								
Response	36	31	30	30	28	30	31	31
Retention	87	88	84	88	85	86	84	86

*Notes. *Dashes indicate that no invitations were sent on that day

^a^Grand mean across studies based on 25,582 and 9135 repeated observations for response and retention, respectively

Table 1 presents an overview of the characteristics of the five studies and overall sample statistics. The field period for all studies was 1 week, and panel members were always randomly assigned to a day of the week. The invitation included brief information upon the study’s topic and the estimated duration of participation. Note that the samples differ in two respects. First, not all studies used all 7 days of the week. For example, we invited panel members on 5 days in Study 1. Likewise, server problems on Sunday in Study 3 resulted in sending invitations scheduled for Sunday on Monday, doubling the sample size on Monday. Only in the two largest Studies 4 and 5 did we invite panel members on all 7 days. Second, in Study 3, we invited participants in a week that included a bank holiday in some federal states of Germany but not in others. Specifically, we invited to Study 3 in the first week of January following New Year's Day, in which Friday, Jan 6 was a bank holiday (i.e., Epiphany) in some federal states.

The two dichotomous dependent variables were response (0 = refused, 1 = responded) and retention (0 = dropped-out, 1 = retained). For robustness and moderator analyses, we took participants’ age (year of birth), education (1 = no degree, 2 = 9 years of schooling, 3 = 10 years of schooling, 4 = 13 years of schooling, 5 = university degree, 6 = PhD), gender (0 = male, 1 = female), and employment status (0 = working, 1 = not working, e.g., students, homemakers, unemployed, or retired persons) from the panel’s database.

### Analyses

As we conducted all five experiments in the same online panel, some panel members were invited to several studies. This results in a two-level hierarchical design, with participants nested within studies. We used generalized estimation equations (GEEs, Ballinger, 2004; Hu et al., 1998; Zeger et al., 1988) to analyze our data. GEEs account for the nested structure by estimating within-subject correlations across measurements, even though these correlations are not modeled explicitly, but rather treated as “nuisance”, which is of no substantive interest (Zeger et al., 1988). GEEs provide estimates of population average effects, that is, the estimated coefficients represent the effect of change for an average individual in the population (Hu et al., 1998; Zeger et al., 1988). An alternative modeling strategy is multilevel modelling (Raudenbush & Bryk, 2003). However, in contrast to GEEs, multilevel models provide unit- or subject-specific parameter estimates, where coefficients represent the effect of a predictor for a specific group or individual (Hu et al., 1998; Raudenbush & Bryk, 2003). In multilevel models, the within-subjects correlation is of substantive interest and is often explained by higher-level predictors. For example, in growth curve models, the change of the dependent variable across time is of interest and often explicitly modeled. As our focus is on the average effect of the day of the week on the dependent variables rather than change within individuals, we chose GEEs to analyze the data using R and the *geepack* package (Halekoh et al., 2006).

To examine the effect of day of the week on response and retention, we estimated several nested models. Because we also conducted sensitivity and exploratory analyses (as explained in the following paragraphs), the large number of models precludes the depiction of all models, many of which did not provide an improvement of model fit. We therefore provide brief descriptions of models and their fit in an online supplement, Tables S1 and S2, for response and retention, respectively.

We started with simple models that included categorical indicators for effects of the study and day of the week. We employed weighted effects coding, using the *wec* package to model effects of study number and day of the week (Nieuwenhuis et al., 2017; te Grotenhuis et al., 2017). In contrast to categorical coding, this coding strategy takes differences in sample size across categories (e.g., studies, days) into account and provides more readily interpretable parameters, because the intercept of the model represents the weighted mean across all five samples of our dependent variable. Weighted effects represent deviations from this full sample’s mean. Also, using another set of weighted effects codes with a different reference category does not change parameters estimate of the remaining categories; it is thus viable to depict parameters for the reference category from additional analyses (te Grotenhuis et al., 2017).

In line with our theoretical reasoning, we also examined whether day of the week effects follow particular trends. Specifically, we used a categorical indicator representing the weekend and additionally modeled linear, quadratic, and cubic trends for the days of the workweek (Mo–Fri). These models closely reflect the substantive trend of fatigue (i.e., linear decline from Monday to Friday), and mood (i.e., quadratic trend with a low at midweek), and a cubic trend might reflect complex interactions among these factors. In addition, we examined whether an identified trend holds across the holiday vs. no holiday condition. Finally, we conducted two sets of additional, exploratory analyses. First, we examined the robustness of our findings when entering control variables (i.e., gender, age, education, employment status) that we obtained from the panel’s database. Second, following Lindgren et al. (2020), we examined potential moderating effects of the day of the week and control variables. Significant effects would indicate that scholars need to take differences across target populations into account.

As our dependent variables are dichotomous, we used a logit link function and a binomial probability distribution, similar to ordinary logistic regression analysis. We followed modeling recommendations by Hin and Wang (2009). These authors recommend selecting variables in a first step using an identity working correlation structure and to examine the suitability of the working correlation structure in a second step. As GEEs use quasi-likelihood estimation procedures, it is not tenable to compare the fit of nested models by means of a likelihood ratio statistic (Heck et al., 2012). As suggested by Hin and Wang (2009), we rely on the QIC measure (Pan, 2001) to select variables for inclusion in the models, with lower QIC values indicating better models in relation to the number of parameters. Note that alternative indices, such as QICC or QICu, resulted in similar findings and are therefore not reported. To choose a working correlation structure, we rely on the CIC (Hin & Wang, 2009). To examine the significance of parameters we used a significance value of 5%.

In gauging the power of our study, we acknowledge that power calculations for GEEs are complex and resort to a simple conservative estimate using logistic regression assuming only one observation per individual, which is *N* = 12,876 instead of 29,592 observations (i.e., assuming within-subject correlations of 1). In addition, definitions of effect sizes using odd ratios depend on the proportion assumed under the null hypothesis (Buchner et al., 2019; Chen et al., 2010). According to a personal communication of the panel administrators, the average response rate in the panel at hand is *p* = 0.43 or 43%. Using this figure as a baseline, an odds ratio of *OR* = 1.37 or 0.73 represents a small effect (Chen et al., 2010), amounting to an increase to *p* = 0.51 or 51%. Using G*Power (Buchner et al., 2019) with *OR* = 1.37, α = .05 (two-tailed), *p* = 0.43, a proportion for our independent variable of *pr* = 1/7 days ≈ 0.14, and *R*^*2*^ = 0.10 for other predictors, we would need a sample of 2941 persons for a power of 1 – β = 0.80. Moreover, as we believe an even smaller increase of the assumed response rate (e.g., Rosenthal & DiMatteo, 2001), for example, from 43 to 46% might be considered valuable, we also estimated the power for the resulting *OR* = 1.13. Using our conservative estimate for *N* = 12,876 yields 1 – β = 0.62. Given that within-subject correlations are most likely below one, we assume that our study has adequate power to detect small and potentially meaningful differences. Similarly, the average retention in the panel amounted to *p* = 0.85. Ignoring repeated observations, for *N* = 5218 the power to detect a small effect (*OR* = 1.46, or an increase to *p* = 0.89) is 1 – β = 0.83.

## Results

Table 2 shows response and retention rates for the five experiments across the 7 days of the week. In all five studies, Monday was equal to or higher than the mean response day of a particular study, and Thursday was at or below the mean. For the other days, the response rate varied, and was higher as well as lower than the average response rate. The maximum difference in response rates between Wednesday and Thursday in the no holiday condition of Study 3 amounts to 12%. Our overall response rate of 31% yields a total of 9135 observations on retention from 5218 participants. With the exception of Study 5, retention rates were highly similar for the days of the week.

Table 3 shows relevant findings from generalized estimation equations (GEEs) for response rates (for information on additional models see Table S1 in the online supplement). Model 1 includes an intercept and four weighted effects to distinguish the experiments. Note that we also depict the weighted effect for the reference category here. Due to weighted effects coding, the intercept represents the overall mean response rate corrected for the correlation within subjects (i.e., exp(– 0.86)/(1+exp(– 0.86)) = 0.30). As depicted in Table 3, all effects are significant, indicating differences in response rates across studies. These might be due to other differences between studies, such as the use of incentives (Studies 3 and 4, see e.g., Göritz, 2006) or possibly the topic of the study. In Model 2, we add six weighted effects that represent the day-of-the-week effect. The QIC indicates better model fit, providing support for a day-of-the-week effect. Because we use weighted effects coding, parameters represent differences from the overall mean. In comparison to the mean, response rates are significantly higher on Monday, *b* = 0.08, *SE* = 0.03, *p* < .001, *OR* = 1.08. and significantly lower on Friday *b* = – 0.09, *SE* = 0.03, *p* < .001, *OR* = 0.86.

**Table 3:** Generalized estimation equations analyzing the effect of day of the week on response rate

Parameter	Model 1 *b* (*SE*)	Model 2 *b* (*SE*)	Model 3 *b* (*SE*)	Model 4 *b* (*SE*)	Model 5 *b* (*SE*)	Model 6 *b* (*SE*)	Model 7 *b* (*SE*)	Model 8 *b* (*SE*)	Model 9 *b* (*SE*)
Intercept	– 0.86 (.02)**	– 0.86 (.02)**	– 0.86 (.01)**	– 0.86 (.02)**	– 0.86 (.02)**	– 0.86 (.02)**	– 0.88 (.02)**	– 0.88 (.02)**	– 0.88 (.02)**
Study									
Study 1 (ref.)^a^	1.07 (.04)**								
Study 2	2.47 (.10)**	2.46 (.10)**	2.46 (.10)**	2.46 (.10)**	2.46 (.10)**	2.47 (.10)**	2.75 (.10)**	2.75 (.10)**	2.75 (.10)**
Study 3	1.00 (.04)**	0.99 (.04)**	0.99 (.04)**	0.99 (.04)**	0.99 (.05)**	0.99 (.05)**	1.20 (.04)**	1.20 (.04)**	1.20 (.04)**
Study 4	– 0.20 (.01)**	– 0.20 (.01)**	– 0.20 (.01)**	– 0.20 (.01)**	– 0.20 (.01)**	– 0.20 (.01)**	– 0.25 (.01)**	– 0.25 (.01)**	– 0.25 (.01)**
Study 5	– 0.45 (.01)**	– 0.45 (.01)**	– 0.45 (.01)**	– 0.45 (.01)**	– 0.45 (.01)**	– 0.45 (.01)**	– 0.51 (01)**	– 0.51 (01)**	– 0.51 (01)**
Day of the week^c^									
Monday		0.08 (.03)**							
Tuesday		0.05 (.03)							
Wednesday		– 0.04 (.03)							
Thursday		– 0.05 (.03)							
Friday		– 0.09 (.04)*							
Saturday		0.03 (.04)							
Sunday (ref.)^a^		0.01 (.03)							
Weekend			– 0.07 (.03)*	– 0.09 (.05)	– 0.07 (.03)*	– 0.07 (.03)*	– 0.07 (.03)*	– 0.07 (.03)*	– 0.07 (.03)*
Day of workweek (linear trend)			– 0.04 (.01)**	– 0.07 (.06)	– 0.04 (.01)**	– 0.04 (.01)**	– 0.04 (.01)**	– 0.04 (.01)**	– 0.04 (.01)**
Day of workweek (squared trend)				0.01 (.01)					
Holiday					– 0.00 (0.07)	– 0.00 (.07)			
Holiday * Weekend						0.12 (.16)			
Holiday * workweek (linear)						0.06 (.07)			
Employment							– 0.03 (.02)	– 0.03 (.02)	– 0.03 (.02)
Education							0.12 (.02)**	0.12 (.02)**	0.12 (.02)**
Gender							– 0.03 (.02)	– 0.03 (.02)	– 0.03 (.02)
Age							0.35 (.02)**	0.35 (.02)**	0.35 (.02)**
Not employed * weekend								– .01 (.04)	
Not employed * day of workweek (linear trend)								.02 (.01)	.02 (.01)*
Quasi-likelihood	– 16864.00	– 16855.00	– 16856.03	– 16855.92	– 16856.03	– 16855.60	– 16451.1	– 16448.5	– 16448.6
QIC	33738.00	33733.00	33726.03	33727.81	33728.03	33731.16	32929.8	32928.7	32926.8

Note. *N* = 12,876 using 29,592 observations for Models 1 to 6, and *N* = 12,845 using 29,526 observations in models 7 to 9 due to missing data in employment variable. All models use an independent working correlation structure. Effects represent weighted effects (i.e., deviations from overall sample mean) for categorical variables. Continuous variables were centered. Employment status (0 = *working*, 1 = *not working*), Gender (0 = *male*, 1 = *female*), day of the week (0 = *no*, 1 = *yes*, reference category is Monday), weekend (0 = *weekday*, 1 = *weekend*), and holiday (0 = *no holiday*, 1 = *holiday*)

^a^The values for the reference categories were calculated in additional models and added to the table (Nieuwenhuis et al., 2017). Since weighted effects represent deviations from the overall sample mean, the values of other effects do not depend upon choice of the reference category

^*^p < .05. ^**^p < .01

To further examine the day-of-the-week effect, we used additional models to test whether it follows a linear (Model 3) or quadratic (Model 4) trend. Specifically, we entered an effect for the weekend and linear and quadratic effects for the days of the workweek. Model 3 exhibited a reduction in QIC and provides evidence for a linear decline of response rates from Monday to Friday, *b* = – 0.04, *SE* = 0.01 *p* < .001, *OR* = 0.96. In addition, compared to the overall response rate, response rates are significantly lower on the weekend, *b* = – 0.07, *SE* = 0.03 *p* = .016, *OR* = 0.91. Model 4 with an additional quadratic effect did not improve model fit as indicated by a higher QIC. Likewise, a model with cubic effects did not provide better fit (see Table S1).

In several additional models, we examined the effects of the bank holiday on response. In Model 5, we entered a main effect of the bank holiday, which did not improve model fit in comparison to the linear trend in Model 3. Also, modeling separate effects for weekend and the workweek in the holiday and no holiday conditions did not yield any improvement, as indicated by the QIC. In sum, Model 3 provided the best fit to the data. Checking the working correlation structure for this model using the CIC showed that an independent working correlation structure fit the data best (cf. Table S1).

In further analyses, we examined the sensitivity of these findings when entering control variables and explored potential moderating effects of controls in line with Lindgren et al. (2020). We found that control variables did exhibit significant main effects on response, but these did not change our substantive findings. Specifically, Model 7 shows that older persons and those with higher education were more likely to respond. Employment and gender did not exhibit any significant effects. With regard to moderating effects of controls regarding the relationship between the day of the week and response rates, we found that only employment moderated this relationship. We did not find any moderating effects for gender, education, and age, using age either a continuous variable or as a trichotomous variable in line with Lindgren et al. (2020 see model descriptions and fit in online supplement, Table S1.

The moderating effect of employment is shown in Models 8 and 9 of Table 3, with Model 9 providing a better fit according to the QIC. In Model 8, neither the effect for weekend nor the workweek trend in the non-employment condition are significant. Model 9 only takes the linear trend over the workweek into account and indicates that the decline over the workweek held only for employed persons and is attenuated by a significantly positive effect for nonemployed persons. This also holds for different parameterizations of the linear trend: when estimating the interaction modeling separate linear trends, we found a significant decline for employed observations, *b* = – 0.06, *SE* = 0.02 *p* < .001, *OR* = 0.95, and an insignificant decline for nonemployed observations, *b* = – 0.03, *SE* = 0.02 *p* = .112, *OR* = 0.97. Figure 1 shows the effect using empirical response rates for observations with and without employment. While a linear trend from Monday to Friday is visible, this trend is less clear for nonemployed persons, who also exhibit high response rates on Monday and Tuesday, but show little variation on the remaining days of the week.

Table 4 shows results from GEEs for retention using the same modeling strategy as before (for information on additional models see online supplement Table S2). In Model 1, we included an intercept and study effects, which indicated significant difference in retention rates between studies. In Model 2, we added six weighted effects that represent the day-of-the-week effect. Although retention was significantly lower on Wednesday, this model fit worse than Model 1 according to the QIC. Similarly, Models 3 and 4 representing linear and squared effects across the workweek did not yield better fit than Model 1 with study effects only. Model 5 includes an effect of the holiday condition, which also did not yield better fit. We did find an effect of the day of the week in the holiday condition, according to Model 6. This model includes a linear effect of weekdays for the holiday condition that was significant, *b* = 0.30, *SE* = 0.12, *p* = .006, *OR* = 1.35, indicating an increase in retention from Monday to Friday. From Table 2, it is evident that this effect is due to higher retention rates on Thursday and Friday in Study 3. To check the robustness of this finding, we added age, education, gender, and employment status as controls in Model 7. The linear effect in the holiday condition remained significant and of the controls, only gender was significant, indicating that men exhibited lower retention rates, *b* = – 0.14, *SE* = 0.04, *p* = .004, *OR* = 0.87. We also examined several exploratory models that examined interactions of control variables with daily models (see Table S2 in the online supplement). No model provided better fit. In sum, there was an increase in retention when the workweek ended with a holiday, but no support for a day-of-the-week effect on retention in regular workweeks.

**Table 4:** Generalized estimation equations analyzing the effect of day of the week on retention rate

Parameter	Model 1 *b* (*SE*)	Model 2 *b* (*SE*)	Model 3 *b* (*SE*)	Model 4 *b* (*SE*)	Model 5 *b* (*SE*)	Model 6 *b* (*SE*)	Model 7
Intercept	2.49 (.06)**	2.49 (.6)**	2.48 (.06)**	2.49 (.06)**	2.49 (.06)**	2.50 (.06)**	2.50 (.06)**
Study							
Study 1 (ref.)^a^	– 0.58 (.09)**						
Study 2	– 1.34 (.11)**	– 1.34 (.11)**	– 1.33 (.11)**	– 1.36 (.11)**	– 1.31 (.11)**	– 1.31 (.11)**	– 1.27 (.11)**
Study 3	0.29 (.10)**	0.29 (.11)**	0.29 (11)**	0.28 (.11)**	0.16 (.13)**	0.16 (.14)**	0.21 (.14)**
Study 4	1.84 (.11)**	1.85 (.11)**	1.85 (.11)**	1.85 (.11)**	1.87 (.11)**	1.87 (.11)**	1.85 (.11)**
Study 5	– 1.74 (.07)**	– 1.74 (.07)**	– 1.74 (.07)**	– 1.74 (.07)**	– 1.72 (.07)**	– 1.71 (.07)**	– 1.74 (.07)**
Day of the week							
Monday		0.03 (.07)					
Tuesday		0.13 (.08)					
Wednesday		– 0.19 (.08)*					
Thursday		0.06 (0.09)					
Friday		0.03 (.09)					
Saturday		– 0.05 (.09)					
Sunday(ref.)^a^		– 0.02 (.08)					
Weekend			– 0.05 (.08)	– 0.16 (.14)		– 0.06 (.08)	– 0.05 (.08)
Day of workweek (linear trend)			– 0.01 (.03)	– 0.15 (.14)		0.01 (.03)	0.01 (.02)
Day of workweek (squared trend)				0.02 (.02)			
Holiday					0.29 (.20)	0.38 (.21)	0.36 (.21)
Holiday * Weekend						0.15 (.40)	0.12 (.40)
Holiday * workweek (linear)						0.30 (.12)*	0.29 (.12)*
Age							0.06 (.04)
Gender							– 0.14 (.04)**
employment							0.07 (.04)
Education							0.02 (.04)
Quasi-likelihood	– 3022.0	– 3017.0	– 3021.5	– 3021.0	– 3021.0	– 3016.3	– 2999.1
QIC	6053.0	6057.0	6056.9	6058.0	6053.0	6052.3	6026.4

Note. *N* = 5218 using 9135 observations for Models 1 to 6, and *N* = 5210 using 9122 observations for Model 7 due to missing control variables. All models use an independent working correlation structure. Effects represent weighted effects (i.e., deviations from overall sample mean) for categorical variables. Continuous variables were centered. Employment status (0 = *working*, 1 = *not working*), Gender (0 = *male*, 1 = *female*), day of the week (0 = *no*, 1 = *yes*, reference category is Monday), weekend (0 = *weekday*, 1 = *weekend*), and holiday (0 = *no holiday*, 1 = *holiday*)

^a^The values for the reference categories were calculated in additional models and added to the table (Nieuwenhuis et al., 2017). Since weighted effects represent deviations from the overall sample mean, the values of other effects do not depend upon choice of the reference category

^*^p < .05. ^**^p < .01

## Discussion

The five experiments provide evidence of a day-of-week effect on the response rate in online access panels. Specifically, response rates decline during the workweek with a high on Monday and a low on Friday. Overall, our findings support our assumption that web survey participation is higher in the beginning of the week. This finding further corroborates several studies that also found higher response rates at the beginning of the week (Faught et al., 2004; Lewis & Hess, 2017; Shinn et al., 2007). The linear decline of response over weekdays is in line with a fatigue account that predicts a linear change across the workweek as opposed to a mood account that would predict a curvilinear (i.e., u-shaped) effect. However, as we did not assess fatigue or mood, this assumption remains to be tested. For example, escapism and the underlying motives (e.g., Hastall, 2017) might cause similar trends. Good days for inviting participants are Monday and Tuesday, with Monday being the best day. We therefore recommend sending invitations at the beginning of the week.

Our exploratory analyses—owing to recommendations of a reviewer and Lindgren et al. (2020)—showed that this effect is restricted to working members of the panel at hand. With working participants, response rates varied with a high on Monday (37%) to a low on Friday (27%). Panel administrators should thus be aware that the day of the week matters more to working participants. With regard to the heterogeneity of prior findings concerning day-of-the-week effects, the percentage of working participants might have affected these findings. In fact, reconsidering Lindgren et al.’s (2020) Table 4, we do find a somewhat similar effect for employed participants, where response rates decline from Monday (48.2%) to Thursday (45.8%), but, contrary to our findings, are higher on Friday (50.2%).

We acknowledge that the effect size for the linear trend (*OR* = 0.96) does not reach a customary definition of a small effect (*OR* = 0.73), and thus this and other findings of this study might appear unimportant. Yet, in line with Rosenthal and DiMatteo (2001), we argue that circumstances should be considered: Because changes in the day of the week come at no costs, this difference might well appeal to panel administrators. For example, using Model 9 of Table 3 and a hypothetical study with our grand mean response rate of 31%, the predicted differences in response rates between Monday and Friday are 4.7 percentage points for employed people and 1.9 percentage points for nonemployed people. Also note that the effect of OR = 0.96 represents a linear trend over days that amounts to *OR* = 0.81 for Friday (i.e., 0.96^5^). In further gauging the practical importance of this finding, we can compare this improvement to the *OR* of 1.19 reported for incentives over no incentives in web-based studies in Göritz’s (2006) meta-analysis. If the present study’s grand mean response rate of 31% represented a condition without incentive, an incentive would yield an estimated response rate of 34.8%, that is, an increase of 3.8 percentage points. This comparison shows that the day-of-the week effect is similar in size to other influences on response rates in online studies.

We found limited evidence for an effect of weekdays on retention. Retention as a dependent variable covers a later stage of the participant journey than response as a dependent variable. Retention merely acts on those participants who have moved to the first page of the study and thus have already incorporated most influences such as mood or work pressure. Only those who had a holiday at the end of the week showed an increase in retention on Thursday and Friday. This finding is again in line with a social construal of time. Note however, that since we did not find any systematic differences in retention in regular workweeks, the practical implications of this effect appear limited.

The present studies are limited in their strong bind to culture. As we argue that social construal affects people's willingness to participate in web-based studies, effects may differ in cultures where other social constructs are in effect. The studies integrated in this work use samples from Germany, which is part of a first-world culture with a Christian tradition. For example, in cultures with a Jewish or Islamic background, Sunday does not belong to the weekend as in Christian cultures, therefore effects may differ or shift to different days (for mood effects see e.g., Tsai, 2019). Moreover, despite sharing a cultural background, there are more subtle differences among Western countries that might render our result idiosyncratic to Germany. Even within a country subcultures exist that might moderate effects (Recabarren et al., 2008). Due to different work schedules and activity patterns, different population strata and professional groups are likely to experience the structure of the week differently (e.g., priests, professionals in sports or artists might be more likely to work on weekends). However, these subcultures are unlikely to affect our results, as we randomly assigned participants to experimental conditions. Yet, we recommend further research in different cultures, countries, and subcultures to explore the boundary conditions of the observed day-of-invitation effect.

Although our sample was not representative of the German online population in a strict statistical sense, we are confident that the results are not idiosyncratic to the experiments reported in this article. First, we examined large, diversely recruited and demographically heterogeneous samples repeatedly and found consistent results. Furthermore, the five studies featured different topics, field times, rewards, and completion times. This might be considered a limitation, because we did not hold these factors constant across studies and this may provide alternative explanations for our findings. However, we believe that any such explanation would have to take several study specifics into account thereby limiting its plausibility. Moreover, invitees in the samples had different levels of motivation to take part in a study. The samples' different levels of motivation are apparent in the studies' total response rates, which vary between 21% in Study 5 and 84% in Study 2. Despite these differences among the studies, the basic pattern that the beginning of the week is favorable for sending out invitations was found throughout. This suggests that differences between studies most likely introduce error variance into our analyses. We think the robustness of our findings supports the external validity of our findings.

Future research may address whether there is an interaction between the day of invitation and the content of the invitation. On certain days, invitees might be more persuaded by particular messages contained in the invitation e-mail than on others (Wilson & Lu, 2008). Another issue for future research is mediation. More elucidation is needed of the presumed mediating role, and relative importance, of causal factors such as mood, fatigue, effort expenditure, and pleasure-seeking. While the issue of underlying mechanisms might not be the foremost concern of survey researchers who are interested in which day of the week they can send out invitations to obtain the highest response rate, throwing light on underlying processes will enable us to make better predictions of which days of the week are good days for inviting a particular clientele. Empirical evidence on fatigue points to more complex processes that may involve anticipation or transition effects (e.g., Weigelt et al., 2021) that we were unable to disentangle. Furthermore, it is an interesting question of whether the day-of-invitation effect extends to a day-of-reminder effect (Göritz & Crutzen, 2012; Göritz, 2014a). If this proven to be the case, to maximize response, not only should panelists be invited at the beginning of the week, but tardy invitees should be reminded of study participation at the beginning rather than at the end of the workweek, too.

In the five experiments at hand, we did not find any effects on retention in regular workweeks without a holiday. For these weeks, the influence of the day of invitation on participation is fully absorbed by the response variable, which antecedes retention. However, the linear increase in the holiday condition shows that retention is affected by additional variables. Perhaps studies that rely on samples with a very low level of motivation to take part in web-based surveys or studies that rely on questionnaires that take much longer to complete than each of the five questionnaires that were used in this work might bring to light any day-of-invitation effect on retention after all.

In this paper, focused on obtainable data quantity, namely response and retention as dependent variables. As an outlook on future research, facets of response quality such as item omissions, the length of answers to open-ended questions, or acquiescence are interesting to examine as additional dependent variables, as there might be trade-offs with data quantity. With regard to the present findings, we recommend those who finish programming their online study on a Thursday or Friday to think about sending out invitations for participation only after the weekend.

### Open practice statement

Data and R syntax are available from the open science framework at https://osf.io/nv689. None of the experiments was preregistered.

## Supplementary information

ESM 1 (DOCX 30 kb)
