# Supplementary material for: The day-of-invitation effect on participation in web-based studies
Source: Behav Res Methods. 2021 Oct 28;54(4):1841–53. doi: 10.3758/s13428-021-01716-0 (PMC9374600; doi:10.3758/s13428-021-01716-0)
Supplement: Supplementary file 1 — (DOCX 30 kb) [file 13428_2021_1716_MOESM1_ESM.docx]

Running Head: Day of invitation

The day-of-invitation effect on participation in web-based studies

Online supplement

*Data availability*

Data and R syntax are available from the open science framework at <https://osf.io/nv689/?view_only=02a14b0fd7c14338a7d1dbc7947a0127>

**Supplement**

Table S1

*Overview of fit indices for all models.*

| Brief Model Description | Quasi-Likelihood | No. of Para-meters | QIC | QICu | CIC |
| --- | --- | --- | --- | --- | --- |
| Models without control variables (*N* = 12876, 29592 observations) |  |  |  |  |  |
| 1: Intercept only (Null model) | -18289.5 | 1 | 36582.3 | 36581.0 | 1.7 |
| 2: Study number | -16864.0 | 5 | 33738.0 | 33738.0 | 5.0 |
| 3: Study number, Day of the week (categorical) | -16855.0 | 11 | 33733.0 | 33733.0 | 11.0 |
| Examining weekly trends |  |  |  |  |  |
| **4: Study number, weekend (categorical), day of work week (linear trend)** | **-16856.0** | **7** | **33726.0** | **33726.1** | **7.0** |
| 5: Study number, weekend (categorical), day of work week (linear and squared trend) | -16855.9 | 8 | 33727.8 | 33727.9 | 8.0 |
| 6: Study number, weekend (categorical), day of work week (linear, squared, and cubic trend) | -16855.9 | 9 | 33729.8 | 33729.8 | 9.0 |
| Examining the working correlation structure of Model 4 |  |  |  |  |  |
| 7: Model 4 with autoregressive working correlation structure (AR1) | -16889.9 | 7 | 33794.0 | 33793.7 | 7.1 |
| 8: Model 4 with exchangeable working correlation structure | -16888.7 | 7 | 33791.7 | 33791.5 | 7.1 |
| 9: Model 4 with unstructured working correlation structure | -16883.2 | 7 | 33781.2 | 33780.3 | 7.5 |
| Examining the holiday condition |  |  |  |  |  |
| 10: Model 4, adding effect for holiday condition (categorical) | -16856.0 | 8 | 33728.0 | 33728.1 | 8.0 |
| 11: Study, weekend (categorical), day of work week (linear trend), holiday condition (categorical), weekend*holiday, work week linear trend*holiday | -16855.6 | 10 | 33731.2 | 33731.2 | 10.0 |
| Models with Control variables (*N* = 12845, 29526 observations) |  |  |  |  |  |
| 12: Study, weekend (categorical), day of work week (linear trend) – similar to model 4 (as baseline without controls) | -16804.0 | 7 | 33622.0 | 33622.0 | 7.0 |
| **13: Model 12 adding controls: not employed (categorical), education, gender, age** | **-16451.1** | **11** | **32929.8** | **32924.2** | **13.8** |
| Exploratory analyses: Control variables as Moderators, using Day of the week (categorical) |  |  |  |  |  |
| 14: Study, Day of the week (categorical) as a baseline to examine effects of control variables again | 16803.0 | 11 | 33629.0 | 33629.0 | 11.0 |
| 15: Study, Control variables (education, employment, age, gender); Baseline model to check whether adding day of the week has effect over and above control variables | -16458.7 | 9 | 32941.1 | 32935.5 | 11.8 |
| 16: Study, Day of the week (categorical), control variables (education, employment, age, gender) | -16450.5 | 15 | 32936.6 | 32931.0 | 17.8 |
| 17: Model 16, adding gender*day of the week | -16447.4 | 21 | 32942.6 | 32936.8 | 23.9 |
| 18: Model 16, adding education*day of the week | -16448.4 | 21 | 32944.6 | 32938.7 | 23.9 |
| 19: Model 16, adding age*day of the week | -16447.0 | 21 | 32943.0 | 32937.0 | 24.0 |
| **20: Model 16, adding employment*day of the week** | **-16443.7** | **21** | **32935.1** | **32929.5** | **23.8** |
| Moderator analyses using linear trend across work week (Baseline: Model 13) |  |  |  |  |  |
| 21: Model 13, adding gender*weekend, gender*day of work week (linear trend) | -16449.1 | 13 | 32929.8 | 32924.2 | 15.8 |
| 22: Model 13, adding education*weekend, education*day of work week (linear trend) | -16450.8 | 13 | 32933.3 | 32927.6 | 15.8 |
| 23: Model 13 adding age*weekend, age*day of work week (linear trend) | -16449.4 | 13 | 32930.6 | 32924.9 | 15.9 |
| **24: Model 13, adding employment*weekend, employment*day of work week (linear trend)** | **-16448.5** | **13** | **32928.7** | **32923.1** | **15.8** |
| 25: Model 13, adding employment* weekend, day of work week (linear trend) | -16449.5 | 12 | 32928.5 | 32922.9 | 14.8 |
| **26: Model 13, adding weekend, employment*day of work week (linear trend)** | **-16448.6** | **12** | **32926.8** | **32921.9** | **14.8** |
| 27: Study, control variables, weekend, day of work week (linear and squared trend), employment*weekend, employment*day of work week (linear and squared trend) | -16446.6 | 15 | 32928.7 | 32923.1 | 17.8 |
| Models using age trichotomization as in Lindgren et al. (2020) |  |  |  |  |  |
| 28: Study, Day, control variables (gender, employment, education age) with age trichotomized | -16591.3 | 16 | 33221.1 | 33214.6 | 19.2 |
| 29: Model 28, adding day*age (trichotomous) | -16584.1 | 28 | 33230.7 | 33224.1 | 31.3 |
| **30: Study, Weekend, day of work week (linear trend), control variables (gender, employment, education, age) with age trichotomized** | **-16591.9** | **12** | **33214.1** | **33207.7** | **15.2** |
| 31: Model 30, adding weekend*age, day of work week (linear trend)*age, age trichotomized | -16590.4 | 16 | 33219.2 | 33212.7 | 19.2 |
| Exploratory model: Is there a squared trend over the entire week? |  |  |  |  |  |
| 32: Study, control variables (employment, age, gender, education), day of week (linear and squared trend, all seven days) | -16452.8 | 11 | 32933.0 | 32927.5 | 13.8 |
| 33: Model 32, adding employment*day of week (linear and squared trend, all seven days) | -16450.8 | 13 | 32933.0 | 32927.6 | 15.7 |

*Note*. Models in bold fit relatively well according to the QIC within their group of comparable models.

Table S2

Overview of fit indices for all models Retention

| Model | Quasi-Likelihood | parameters | QIC | QICu | CIC |
| --- | --- | --- | --- | --- | --- |
| Models without control variables (*N* = 5.218 persons, 9135 observations) |  |  |  |  |  |
| 1: Intercept only (Null model) | -3677.7 | 1 | 7357.3 | 7357.3 | 0.97 |
| **2: Study number** | **-3022.0** | **5** | **6053** | **6053** | **5.00** |
| 3: Study number, Day | -3017 | 11 | 6057 | 6057 | 11.00 |
| Examining weekly trends |  |  |  |  |  |
| 4: Study number, weekend (categorical), day of work week (linear trend) | -3021.5 | 7 | 6056.9 | 6056.9 | 6.96 |
| 5: Study number, weekend (categorical), day of work week (linear and squared trend) | -3021.0 | 8 | 6058.0 | 6058.0 | 8.00 |
| 6: Study number, weekend (categorical), day of work week (linear, squared, and cubic trend) | -3021.0 | 9 | 6059.0 | 6058.0 | 9.00 |
| Examining the working correlation structure of Model 2 |  |  |  |  |  |
| 7: Model 2 with autoregressive working correlation structure (AR1) | -3022.0 | 5 (CIC = 5) | 6053.0 | 6053.0 | 5.00 |
| 8: Model 2 with exchangeable working correlation structure | -3021.7 | 5 (5.01) | 6053.4 | 6053.4 | 5.01 |
| 9: Model 2 with unstructured working correlation structure | -3021.9 | 5 (5,07) | 6053.9 | 6053.7 | 5.07 |
| Examining the holiday condition |  |  |  |  |  |
| 10: Model 2, adding effect for holiday condition | -3021.0 | 6 | 6053.0 | 6053.0 | 6.00 |
| **11: Study, weekend (categorical), day of work week (linear trend), holiday condition (categorical), weekend*holiday, work week (linear trend)*holiday** | **-3016.3** | **10** | **6052.3** | **6052.6** | **9.87** |
| 12: Study, weekend (categorical), day of work week (linear & squared trend), holiday condition (categorical), weekend*holiday, work week (linear & squared trend)*holiday | -3015.5 | 12 | 6055.2 | 6055.0 | 12.10 |
| 13: Study, weekend (categorical), day of work week (linear, squared, & cubic trend), holiday condition (categorical), weekend*holiday, work week (linear, squared, & cubic trend)*holiday | -3015.0 | 14 | 6058.0 | 6085.0 | 14.00 |
| 14: Study, Day of week (categorical), holiday*day of week (categorical)^a^ | -3011.0 | 17 | 6057.0 | 6057.0 | 17.00 |
| Models with Control variables (*N* = 5210 persons, 9122 observations) |  |  |  |  |  |
| 15: Study, weekend (categorical), day of work week (linear trend), holiday condition (categorical), weekend*holiday, work week linear trend*holiday – (similar to model 11 as baseline without controls) | -3008.3 | 10 | 6036.3 | 6036.6 | 9.87 |
| **16: Model 15 adding controls: not employed (categorical), education, gender, age** | **-2999.1** | **14** | **6026.4** | **6026.2** | **14.10** |
| 17: Study, Controls (a simple model) | -3004.1 | 9 | 6026.8 | 6026.3 | 9.25 |
| Exploratory analysis: Control variables as Moderators, using Day of the week (categorical) |  |  |  |  |  |
| 18: Study, Day of the week (categorical, equivalent to model 3) | -3009.0 | 11 | 6040.0 | 6040.0 | 11.00 |
| 19: Study, Day of the week (dummies), control variables (education, employment, age, gender) | -3000.1 | 15 | 6030.4 | 6030.1 | 15.20 |
| 20: Model 19, adding gender*day of the week | -2995.1 | 21 | 6032.7 | 6032.2 | 21.30 |
| 21: Model 19, adding education*day of the week | -2998.0 | 21 | 6038.6 | 6037.9 | 21.30 |
| 22: Model 19, adding age*day of the week | -2996.4 | 21 | 6035.5 | 6034.9 | 21.30 |
| 23: Model 19, adding employment*day of the week | -2995.6 | 21 | 6033.6 | 6033.1 | 21.30 |
| Moderator analyses using linear trend across work week by holiday interaction (Baseline: Model 15) |  |  |  |  |  |
| 24: Model 15, adding gender*weekend*holiday, gender*day of work week (linear trend) * holiday | -2997.7 | 19 | 6033.2 | 6033.4 | 18.90 |
| 25: Model 15, adding education*weekend*holiday, education*day of work week (linear trend) *holiday | -2995.8 | 19 | 6029.3 | 6029.6 | 18.80 |
| 26: Model 15 adding age*weekend*holiday, age*day of work week (linear trend) *holiday | -2998.0 | 19 | 6035.2 | 6034.0 | 19.60 |
| 27: Model 15, adding employment*weekend*holiday, employment*day of work week (linear trend) *holiday- | -2997.5 | 19 | 6032.5 | 6033.0 | 18.70 |
| Models using age trichotomization as in Lindgren et al. (2020) |  |  |  |  |  |
| 28: Study, Day (categorical), control variables (gender, employment, education age) with age trichotomized | -3000.1 | 16 | 6032.5 | 6032.2 | 16.10 |
| 29: Model 28, adding day(categorical) *age (trichotomous) | -2997.1 | 28 | 6050.5 | 6050.2 | 28.10 |
| 30: Study, Weekend, day of work week (linear trend), control variables (gender, employment, education, age) with age trichotomized | -3004.1 | 12 | 6032.5 | 6032.2 | 12.20 |
| 31: Model 30, adding weekend*age, day of work week (linear trend)*age, age trichotomized | -3003.2 | 16 | 6038.6 | 6038.3 | 16.20 |
| Exploratory model: Is there a squared trend over the entire week? |  |  |  |  |  |
| 32: Study, control variables (employment, age, gender, education), day of week (linear and squared trend, all seven days) | -3003.9 | 11 | 6030.3 | 6029.9 | 11.20 |
| 33: Model 29, adding holiday*day of week (linear and squared trend, all seven days) | -3000.8 | 14 | 6030.4 | 6029.6 | 14.40 |

*Note*. Models in bold fit relatively well according to the QIC within their group of comparable models.

^a^ no invitations were sent on Sunday in the holiday condition.
